# The Effects of Early-Onset Pre-Eclampsia on Placental Creatine Metabolism in the Third Trimester

**DOI:** 10.3390/ijms21030806

**Published:** 2020-01-26

**Authors:** Stacey J. Ellery, Padma Murthi, Paul A. Della Gatta, Anthony K. May, Miranda L. Davies-Tuck, Greg M. Kowalski, Damien L. Callahan, Clinton R. Bruce, Euan M. Wallace, David W. Walker, Hayley Dickinson, Rod J. Snow

**Affiliations:** 1 The Ritchie Centre, Hudson Institute of Medical Research, and Department of Obstetrics & Gynaecology, Monash University, Clayton 3168 Australia; padma.murthi@monash.edu (P.M.); miranda.davies@hudson.org.au (M.L.D.-T.); euan.wallace@monash.edu (E.M.W.);; 2 Department of Pharmacology, Monash University, and Department of Obstetrics and Gynaecology, University of Melbourne, Parkville, Melbourne 3010, Australia; 3 Institute for Physical Activity and Nutrition, School of Exercise and Nutrition Sciences, Deakin University, Geelong 3216, Australia; paul.dellagatta@deakin.edu.au (P.A.D.G.); a.may@deakin.edu.au (A.K.M.); greg.kowalski@deakin.edu.au (G.M.K.); clinton.bruce@deakin.edu.au (C.R.B.); rod.snow@deakin.edu.au (R.J.S.); 4 Centre for Cellular and Molecular Biology, School of Life and Environmental Science, Deakin University, Burwood, Melbourne 3125, Australia; damien.callahan@deakin.edu.au; 5 School of Health & Biomedical Sciences, RMIT University, Melbourne 3082, Australia; david.walker@rmit.edu.au

**Keywords:** placental bioenergetics 1, phosphocreatine 2, metabolism 3, obstetrics 4

## Abstract

Creatine is a metabolite important for cellular energy homeostasis as it provides spatio-temporal adenosine triphosphate (ATP) buffering for cells with fluctuating energy demands. Here, we examined whether placental creatine metabolism was altered in cases of early-onset pre-eclampsia (PE), a condition known to cause placental metabolic dysfunction. We studied third trimester human placentae collected between 27–40 weeks’ gestation from women with early-onset PE (*n* = 20) and gestation-matched normotensive control pregnancies (*n* = 20). Placental total creatine and creatine precursor guanidinoacetate (GAA) content were measured. mRNA expression of the creatine synthesizing enzymes arginine:glycine aminotransferase (*GATM*) and guanidinoacetate methyltransferase (*GAMT*), the creatine transporter (*SLC6A8*), and the creatine kinases (mitochondrial *CKMT1A* & cytosolic *BBCK*) was assessed. Placental protein levels of arginine:glycine aminotransferase (AGAT), GAMT, CKMT1A and BBCK were also determined. Key findings; total creatine content of PE placentae was 38% higher than controls (*p* < 0.01). mRNA expression of *GATM* (*p* < 0.001), *GAMT* (*p* < 0.001), *SLC6A8* (*p* = 0.021) and *BBCK* (*p* < 0.001) was also elevated in PE placentae. No differences in GAA content, nor protein levels of AGAT, GAMT, BBCK or CKMT1A were observed between cohorts. Advancing gestation and birth weight were associated with a down-regulation in placental *GATM* mRNA expression, and a reduction in GAA content, in control placentae. These relationships were absent in PE cases. Our results suggest PE placentae may have an ongoing reliance on the creatine kinase circuit for maintenance of cellular energetics with increased total creatine content and transcriptional changes to creatine synthesizing enzymes and the creatine transporter. Understanding the functional consequences of these changes warrants further investigation.

## 1. Introduction

Pre-eclampsia (PE) is a pregnancy-specific hypertensive disorder that affects about 5% of all pregnancies and is responsible for more than 50,000 maternal deaths each year worldwide [1,2,3,4]. It is hypothesized that the pathophysiology of early-onset pre-eclampsia (<34 weeks’ gestation) has its origins in the placental and uterine vasculature, where inadequate vascular remodeling leads to repeated ischemic-reperfusion (I/R) events within placental tissue [5]. This chronic impaired placental perfusion is thought to promote the production and release of inflammatory and anti-angiogenic factors that are known to be elevated in the maternal circulation of PE cases [6]. These factors damage the maternal vasculature inducing wide-spread endothelial dysfunction, hypertension and, in severe cases, organ injury and maternal death [7].

Impaired oxygen delivery to the placenta also destabilizes placental metabolism and bioenergetics, which in turn limits ATP-dependent processes and promotes the production of reactive oxygen species, often leading to functional insufficiencies in the placenta that can negatively impact the developing fetus. Indeed, the risk of fetal growth restriction and preterm birth, either spontaneous or through iatrogenic delivery, is increased with PE [2,8].

A recent wide-spectrum metabolomic analysis identified increased creatine and creatinine in cord plasma in PE pregnancies [9] indicating that, among other metabolic changes, fetal and potentially placental creatine homeostasis is altered in PE. Creatine—*N*-(aminoiminomethyl)*N*-methyl glycine—is a nitrogenous amino acid derivative obtained from our diet and endogenously synthesized by some tissues, including the placenta [10]. Through a reversible phosphorylation/dephosphorylation reaction catalyzed by mitochondrial (CKMT1A) and cytosolic (BBCK) creatine kinases, creatine stabilizes cellular bioenergetics through both spatial and temporal maintenance of adenosine diphosphate and triphosphate (ADP/ATP) ratios [11].

Our understanding of the importance of creatine provision to the developing placenta and fetus in healthy pregnancies has expanded in recent years [12,13,14,15,16]. We have previously demonstrated that the term human placenta expresses both enzymes (arginine:glycine aminotransferase [AGAT] and guanidinoacetate methyltransferase [GAMT]) required to synthesize creatine *de novo*, and can produce both creatine and the creatine precursor guanidinoacetate (GAA) ex vivo, thus bypassing a complete reliance on dietary creatine or endogenous synthesis by the maternal kidney and liver for its creatine supply [10]. Our further investigations into creatine metabolism in cases of third trimester placental insufficiency and fetal growth restriction in the absence of PE suggest that the compromised placenta adapts by increasing tissue creatine concentrations, which may reflect a heightened reliance on the creatine kinase circuit to stabilize placental bioenergetics under chronic hypoxic conditions [17]. Whether placental metabolic changes associated with PE induce changes in placental creatine metabolism, and potentially creatine transfer to the fetus, is unknown.

This study examined whether early-onset PE altered placental creatine metabolism. Specifically, we assessed placental creatine and GAA content, the expression of the enzymes required for creatine synthesis, creatine intracellular uptake and utilization via the creatine kinase circuit. We hypothesized that PE would have effects on placental creatine homeostasis that would include pathways involved in its synthesis and transport. Our findings are discussed in relation to a possible placental response to minimize PE-induced injury, which could have the dual benefit of reducing the maternal syndrome of PE and improving fetal wellbeing.

## 2. Results

### 2.1. Population Characteristics

Table 1 summarizes the maternal characteristics and pregnancy outcomes for the normotensive control and early-onset PE cohorts. Women with PE were hypertensive (162.3 ± 11.8/102.9 ± 5.9 mmHg) with confirmed proteinuria. These women were younger than controls (*p* < 0.01) and more likely to be nulliparous (*p* < 0.01). There was no significant difference in gestational age between control and PE cohorts. However, infants born from pregnancies complicated by PE had lower birth (*p* < 0.05) and placenta (*p* < 0.05) weights compared to controls.

### 2.2. Placental Creatine and GAA Content

**Figure 1:**
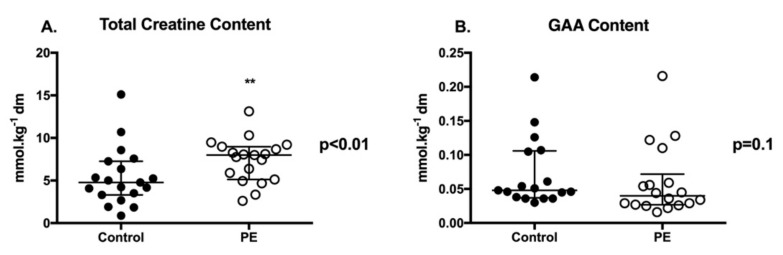
Placental Creatine and Creatine Precursor Guanidinoacetate (GAA) Content. Total creatine (**A**) and GAA (**B**) content of *n* = 19 healthy control (closed circle) and *n* = 19 PE (open circle) placentae. Two sample t-tests were used for statistical comparison. Data are present means ± SD. ** *p* < 0.01.

Total creatine content of PE placentae was significantly higher (+38%) than controls (Figure 1A; *p* < 0.01). Placental content of GAA (creatine precursor) was not significantly different between PE and control cohorts (Figure 1B). Baby sex had no effect on placental creatine or GAA content in either control or PE pregnancies.

### 2.3. Creatine Synthesis and Transport Genes and Proteins

**Figure 2:**
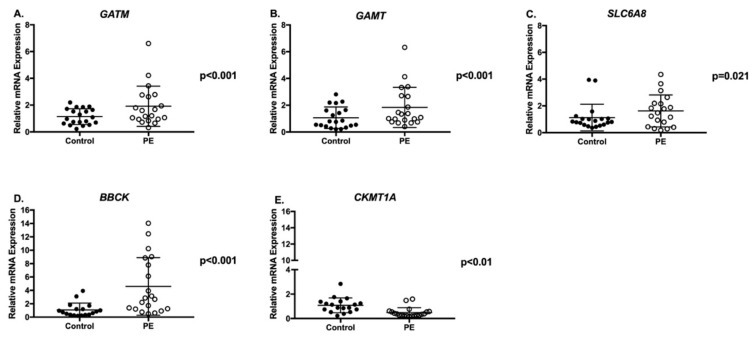
Placental Gene Expression of the Creatine Synthesizing Enzymes, the Creatine Transporter and Kinases. Creatine synthesizing enzymes *GATM* gene that codes for AGAT (**A**) and *GAMT* (**B**), the creatine transporter *SLC6A8* (**C**), cytosolic creatine kinase *BBCK* (**D**) and mitochondrial creatine kinase *CKMT1A* (**E**). *n* = 20 normotensive control (closed circle) and *n* = 20 PE (open circle). Data are presented relative to the control cohort. Values are mean ± SD. Wilcoxon Rank Sum was used for statistical comparison. Significance was set at *p* ≤ 0.025, following a Benjamini-Hochberg adjustment for false-positives.

**Figure 3:**
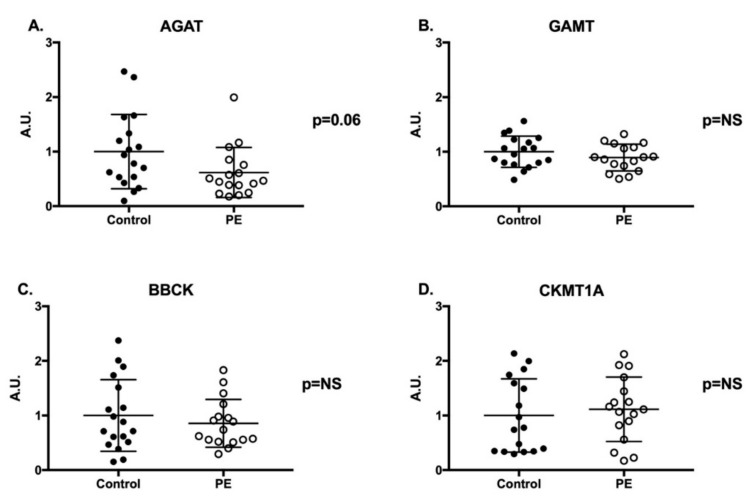
Placental Protein Expression of the Creatine Synthesizing Enzymes and Kinases. Creatine synthesis enzymes AGAT (**A**) and GAMT (**B**), cytosolic creatine kinase BBCK (**C**) and mitochondrial creatine kinase CKMT1A (**D**). *n* = 19 normotensive control (closed circle) and *n* = 19 PE (open circle). Data were normalized to total protein and a control sample run across blots. Data are expressed in arbitrary units (A.U.) relative to the control cohort. Wilcoxon Rank Sum was used for statistical comparison. Significance was set at *p* ≤ 0.025, following a Benjamini-Hochberg adjustment for false-positives. NS: not significant.

*GATM* and *GAMT* mRNA expression were up-regulated 2-fold in PE placentae compared to controls (Figure 2A *p* < 0.001 and Figure 2B *p* < 0.001, respectively). We also observed a two-fold increase in mRNA expression for the creatine transporter (*SLC6A8*) and a 4-fold increase in cytosolic *BBCK* mRNA expression in PE placentae compared to controls (Figure 2C *p* = 0.021 and Figure 2D *p* < 0.001, respectively). Conversely, mitochondrial creatine kinase (*CKMT1A*) mRNA expression was significantly decreased in PE placentae (Figure 2E *p* < 0.01). Despite changes in mRNA expression of the creatine synthesizing enzymes and creatine kinases, there were no significant differences in protein abundance of AGAT, GAMT, BBCK or CKMT1A between the PE and control placentae (Figure 3, blots available in Supplementary Information as Figures S1–S4). Baby sex had no impact on placental gene and protein expression in either control or PE pregnancies.

### 2.4. Correlations between Creatine Metabolism and Pregnancy Outcomes

**Figure 4:**
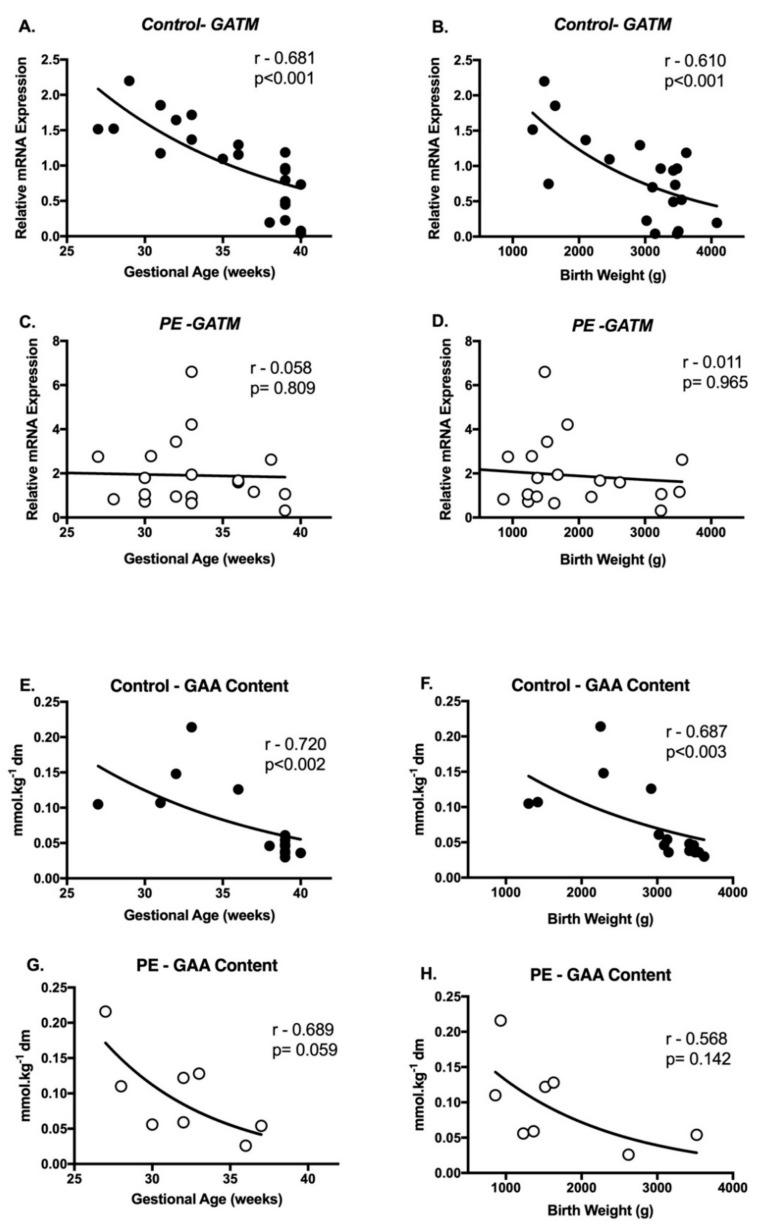
Associations (r) between placental *GATM* mRNA and gestational age (**A**) and birth weight (**B**) in the control cohort; placental *GATM* mRNA and gestational age (**C**) and birth weight (**D**) in the PE cohort; placental GAA content and gestational age (**E**) and birth weight (**F**) in the control cohort and GAA content and gestational age (**G**) and birth weight (**H**) in the PE cohort. *r* values were generated using Spearman’s correlation coefficient. Normotensive placenta data are represented by closed circles and PE placentae open circles. Significance was set at *p* ≤ 0.004, following a Benjamini-Hochberg adjustment for false-positives.

We explored associations between placental metabolite content, gene and protein expression, and then these laboratory measures with maternal characteristics (age, parity, mode of delivery) and pregnancy outcomes (baby sex, gestational age, birth weight and placental weight). There was a significant decrease in placental *GATM* mRNA expression with advancing gestational age (Figure 4A, *r* = −0.681; *p* < 0.001) and birth weight (Figure 4B, *r* = −0.610; *p* < 0.001) in controls. These gene associations were absent in the PE cohort (Figure 4C,D). Similar correlations were observed with placental GAA, with tissue content declining with advancing gestational age (Figure 4E, *r* = −0.720; *p* < 0.002) and birth weight (Figure 4F, *r* = −0.687; *p* < 0.003) in normotensive controls. These associations were again absent in the PE cohort (Figure 4G,H). Placental creatine content was not significantly associated with any other maternal characteristics or pregnancy outcomes. Nor were mRNA expression patterns of *GAMT, SLC6A8, BBCK, CKMT1A* or AGAT, GAMT, BBCK or CKMT1A protein abundance.

## 3. Discussion

In addition to widespread endothelial dysfunction, alterations to maternal, placental and fetal metabolism are hallmarks of PE [18]. In this study, we explored third trimester placental creatine metabolism in early-onset PE and gestation-matched normotensive pregnancies. Our findings confirm the recent metabolomic reports of altered creatine homeostasis with PE [9]; moreover, we identified significant changes in expression of genes associated with the synthesis and transport of creatine within the placenta. Our main finding was that total creatine content of the third trimester PE placentae was significantly higher than controls. Similar adaptations have recently been described in placental insufficiency leading to fetal growth restriction (FGR) in the absence of PE [17] and in pregnancies that occurred at high altitude [19]. To the best of our knowledge, these studies are the first to describe increased creatine content in human tissue exposed to perturbations in oxygen delivery, in vivo.

There are three potential mechanisms for changes in placental creatine content between normotensive and PE placentae. These include changes to creatine to creatinine degradation rates, increased endogenous synthesis and/or increased cellular uptake of creatine from the circulation. It is unlikely that changes to the rate of creatine degradation to creatinine would have led to differences in placental creatine content between cohorts, as this is a spontaneous, non-enzymatically driven process that occurs at a near constant rate of ~1.7% of total body creatine content per day [20]. This leaves changes to endogenous placental creatine synthesis and/or rates of cellular up-take as potential mechanisms for increasing placental creatine content with PE. As cellular metabolism is in a constant state of flux, it is difficult to ascertain exactly which of these processes (de novo synthesis or cellular up-take, or both) resulted in the increased total creatine content of the PE placenta in this retrospective study. However, we make the following observations.

PE up-regulated placental gene expression of the creatine synthesizing enzymes *GATM* and *GAMT*. Modulation of *de novo* creatine synthesis is usually discussed in the context of AGAT (i.e., *GATM* mRNA expression and/or AGAT activity) as this is thought to be the rate-limiting step of creatine biosynthesis [21]. Mediators of AGAT expression and activity include thyroid hormone, growth hormone, and circulating levels of arginine, citrulline and creatine [22,23]. The influence of arginine, citrulline and creatine on the rate of endogenous creatine synthesis has mainly been described as a down-regulation of *GATM* and AGAT when these metabolites are in excess, which is the opposite to what has been observed in the PE placentae [22]. On the contrary, adequate concentrations of thyroid and growth hormone are required to maintain AGAT activity, with studies in thyroidectomy or hypophysectomy rats observing decreased AGAT renal activity [24]. Whether increases in thyroid or growth hormone above basal levels can increase AGAT activity is unknown. There is the potential that increased placental growth factor production by the PE placenta may be influencing these transcriptional pathways, and maintaining the placental *GATM* mRNA expression with advancing gestational age and birth weight observed in the current study, a finding that was absent in normotensive controls [25].

Despite increases in gene expression of both *GATM* and *GAMT* and the increased total creatine content, there were no detectable changes in AGAT or GAMT protein levels, indicating a mismatch in transcription and abundance of the enzymatic machinery of creatine synthesis in the PE placentae. It may be that placental endoplasmic reticulum stress reported with PE may hinder increased production of AGAT and GAMT enzymes [26]. There is also the potential of an increased rate of *de novo* creatine synthesis by increased activity of the AGAT and GAMT enzymes in the absence of changes to overall protein abundance. As no change in placental GAA content was observed between cohorts, we would contend that any changes in AGAT and GAMT activity would have been in equilibrium. We cannot rule out how histomorphological differences between control and PE placentae may have influenced measured AGAT and GAMT expression, as our assessments were conducted on tissue homogenates. An increase in terminal villi density, but an overall decline to villous surface area are characteristic of placental PE histology [27]. AGAT is expressed in stromal and endothelial cells of the fetal capillaries and GAMT in the syncytiotrophoblast of the fetal villi [10], so it is plausible that levels of AGAT and GAMT could be altered by changes to cell populations. However, one would anticipate that the known histological changes within the PE placenta would lead to a down-regulation in expression, not the increases in mRNA expression and no change in protein levels observed in the current cohort.

The alternate route of increasing placental creatine content is by increased transport of creatine into the placental cells via the creatine transporter [28]. This could simply be the result of increased maternal circulating creatine levels with PE. While no studies have characterized changes in maternal creatine levels in PE cases, increased creatine in cord plasma has been reported [9]. The retrospective nature of the current study and lack of maternal blood sampling inhibited our ability to explore this hypothesis further. Assessment of maternal circulating concentrations of creatine should be a focus of future prospective cohort studies. The current study did find a 2-fold increase mRNA expression of the creatine transporter *SLC6A8* in PE placentae compared to controls. A potential mechanistic pathway for increased creatine transporter expression and activity in the placenta is via AMP-activated protein kinase (AMPK), a key regulator of cellular energy metabolism that has been implicated in the pathophysiology of PE [29]. In cardiomyocytes, activation of AMPK pathways has been shown to up-regulate creatine transporter expression and activity, ultimately increasing cellular creatine up-take [30]. This effect is thought to be indirect, as the creatine transporter does not contain substrate sites for AMPK mediated phosphorylation. Potential intermediates are co-activator peroxisome activated receptor γ coactivator-1 alpha (PGC-1α) and estrogen-related receptor α (ERRα), both known to be regulated by AMPK and induce the expression of genes involved in cellular bioenergetics, including mitogenesis [31]. Studies in skeletal muscle cells (L6 myotubes) have described direct interactions between PGC-1α/ERRα, increased creatine transporter mRNA expression and cellular creatine up-take [32]. As there have been mixed reports of increases and decreases to placental mitochondrial content and PGC-1α expression with PE [33,34], further exploration of this mechanistic pathway is required before conclusions about AMPK’s role in driving changes to creatine transport and cellular up-take in PE placentae can be drawn. In vitro techniques should be employed for these analyses to control for changes in placental metabolism associated with tissue collection and processing times.

It is probable that placental mitochondrial dysfunction [8], and thus changes in AMPK expression, are also influencing how the PE placenta is utilizing the available creatine for ATP homeostasis. AMPK is a known regulator of the expression of the cytosolic isoform of creatine kinase *BBCK* (responsible for the hydrolysis of phosphocreatine and phosphorylation of ADP to form ATP) [35]. This finding is consistent with the increased *BBCK* mRNA expression observed in PE placentae in the current study. Activation of the HIF-2α hypoxia response element, which has been shown to be up-regulated in PE, may also contribute to changes in *BBCK* mRNA expression, as activation of HIF-2α enhances BBCK expression in apical intestinal epithelial cells with inflammatory bowel disease [36]. Again, there was no detectable difference in protein abundance in the current study. Thus, further interrogation of these mechanisms is required to elucidate the interactions between metabolic regulatory pathways and creatine metabolism in the PE placenta. Known variations in mitochondrial function and thus capacity to generate ATP between pre-term and term PE placentae should be considered within this context [37].

Interestingly, the mechanism (de novo synthesis and/or cellular uptake) for increasing placental creatine levels may be different between the PE and FGR pathologies. Placentae of growth restricted infants had lowered GAA levels and reduced mRNA expression of *GATM* and *GAMT*, compared to the PE placentae that showed no change in GAA content and an up-regulation in *GATM* and *GAMT* [17]. It is generally accepted that the level of placental insult is higher in PE than in FGR, producing a more substantial burden of placental pro-inflammatory and anti-angiogenic factors and their interactions on maternal endothelium in multiple-organ systems [38]. As maternal kidney injury is a primary manifestation of the PE syndrome, changes to maternal systemic creatine synthesis, mainly GAA production by AGAT activity in the renal proximal tubules, may alter maternal creatine homeostasis, placing a more significant burden on the placenta to maintain adequate creatine levels for placental and fetal requirements.

Increased de novo creatine synthesis, either by the placenta or maternal production by the kidney and liver could have further implications for the pathogenesis of PE. The methylation of GAA by GAMT produces creatine and S-Adenosylhomocysteine, contributing to around 40% of total homocysteine production under basal conditions [39]. Elevated maternal homocysteine levels have been identified in several PE cohort studies. As homocysteine can cause endothelial dysfunction, it is thought that hyperhomocysteinemia could contribute to maternal vascular dysfunction with PE [40,41]. Considering that a consequence of increased *de novo* creatine synthesis in response to PE could be the over-production of homocysteine, understanding these pathways may have implications for the management of the maternal PE syndrome beyond placental bioenergetics.

There were several limitations to this study. Villous tissue was dissected from a central placental cotyledon only, and not randomly sampled from several sites across the placenta, as is now the recommendation for molecular studies [42]. The retrospective nature of this study also meant that some maternal demographic information that may have been relevant (i.e., ethnicity) was not available. Finally, the lack of an appropriate antibody inhibited our ability to quantify creatine transporter protein concentrations, via western blot [28].

## 4. Materials and Methods

### 4.1. Human Research Ethics, Sample Collection and Storage

Archived placental villous samples, from 20 normotensive controls and 20 women with early-onset PE, were collected at the Royal Women’s Hospital Melbourne, Australia for research purposes, and explicitly accessed for this study. The Society of Obstetric Medicine of Australia and New Zealand (SOMANZ) research definition of PE was used to select the PE cohort [43]. Gestation-matched controls were selected from normotensive term pregnancies, idiopathic preterm deliveries or preterm elective deliveries for indications not associated with placental function. All controls were absent of placental pathology and delivered babies with birth weights appropriate for gestational age [44]. The collection and archiving of all samples had the approval of the relevant institutional human research ethics committees in 2000 and 2001 (HREC# 01/46, 00/27). Each woman gave written informed consent for tissue collection and the recording of de-identified demographic information and pregnancy outcomes. Where available, information on maternal age, parity, gestational age, mode of delivery, baby sex, birth weight and placental weight were extracted from electronic records. Sample processing was within 20 min of placental delivery. Villous tissue was dissected from a central placental cotyledon. The decidua was removed before tissues were divided into pieces, snap-frozen in liquid nitrogen and stored at –80 °C for future analysis [45].

### 4.2. Sample Processing

#### 4.2.1. Creatine and GAA Analysis

Snap frozen placental tissues of sufficient weight (*n* = 19 control and *n* = 19 PE) were freeze dried overnight at −80 °C. Powdered samples were then weighed (3–4 mg) and extracted on ice using 0.5 M perchloric acid/1mM Ethylenediaminetetraacetic acid (EDTA) before being neutralized with 2.1 M potassium hydrogen carbonate as previously described [17]. Total creatine content (creatine + phosphocreatine) was measured using fluorometric assays [46]. GAA was measured by liquid chromatography tandem mass spectrometry (LC-MS/MS) through a slight modification of the method of Tran et al. (2014) [47]. Briefly, 10 µL of unlabeled standard, placental tissue lysate or lysis solution (extraction blank) were deproteinized with 200 µL of methanol containing 2.5 µM 2,2-d_2_-GAA (Sigma, Rowville, Victoria, Australia deuterium labelled internal standard. Samples were vortexed for 20 min before centrifugation at 15,000 rpm for 5 min, followed by the transfer of 180 µL of the supernatant into 250 µL glass inserts. Samples were then dried in a speed vacuum prior to being derivatized (butylated) by the addition of 100 µL of 3 M butanol-hydrochloric acid and incubation at 60 °C for 30 min. After derivatization, samples were dried under speed vacuum and the residue re-suspended in 100 µL of methanol: water (1:1 *v/v*). The LC-MS/MS system comprised a vacuum degasser, binary pumps, column oven and a temperature-controlled autosampler (Shimadzu, Nexera^®^ UPLC, Rowville, Victoria, Australia) interfaced with a triple quadrupole mass spectrometer (Shimadzu, LC-MS-8040) with positive electrospray ionization and operated in multiple reaction monitoring (MRM) mode. The MRM transitions for the [M+H^+^]^+^ butylated derivatives were: GAA 174.1 m/z–101.1 m/z, d_2_-GAA 176.1 m/z–103.1 m/z, the collision energy (−15 V) and quad bias voltages were optimized using standards. The LC column was a 2.1 mm × 100 mm, 1.8 µm C18 Zorbax Elipse plus (Agilent, Mulgrave, Victoria, Australia) maintained at 30 °C. The sample and standard injection volume was 5 µL. Samples were eluted with a binary mobile gradient at 0.4 mL/min (mobile phase A water 0.1% formic acid, mobile phase B acetonitrile 0.1% formic acid) with the following program: initial composition was 5% B followed by a linear increase to 50% B over 5 min. The column was then washed at 100% B for 2 min then re-equilibrated at 5% B for 3 min. Total run time was 11 min. GAA eluted at 2.6 min. Peak areas were determined using Lab Solutions Post-run Browser software (Shimadzu). The GAA concentration in the tissue lysate was calculated via linear regression of a serially diluted external (unlabeled) standard series using the isotope dilution technique, after which the concentration was adjusted to the total lysate volume, dilution factor and then normalized to tissue dry mass.

#### 4.2.2. Gene Expression Analysis

Total RNA was extracted from placental tissues (*n* = 20 control and *n* = 20 PE) using RNeasy Kits (Qiagen, Glen Forrest, Australia) and reverse transcribed to form cDNA with SuperScript III Reverse Transcriptase (Invitrogen, ThermoFisher Scientific, Scoresby, Australia), according to manufacturer’s protocol. mRNA expression of the creatine synthesizing enzymes (*GATM*-the gene that expresses AGAT & *GAMT*), the creatine transporter (*SLC6A8*) and creatine kinase isoforms (*BBCK* & *CKMT1A*) was determined using Fluidigm Biomark HD system with TaqMan chemistry. TaqMan probe sequences are detailed in Table 2. *RN18S* was used as the housekeeping gene, after conserved expression between the cohorts was validated. Data from qPCR was analyzed according to the ^ΔΔ^*C*_T_ method [48] and results are expressed relative to the control cohort.

#### 4.2.3. Protein Analysis

The abundance of AGAT, GAMT, BBCK and CKMT1A in placental protein extracts (*n* = 19 control and *n* = 19 PE) was measured by western immunoblotting, as previously described [17]. Briefly, 40 μg protein/sample was separated on 4%–15% Criterion™ TGX Stain-Free™ precast gels in 10× Tris/Glycine/SDS buffer solution (Bio-Rad, Gladesville, Australia) (for sample arrangement see Supplementary Information Tables S1 and S2). Two micrograms of human liver protein extract (Santa Cruz Biotechnologies sc-363766, Dallas, TX, USA) were included on gels for AGAT and GAMT assessment, as a positive control. Gels for creatine kinase B type and creatine kinase u-type mitochondrial assessment included 2 μg of human brain protein extract (Santa Cruz Biotechnologies sc-364375, Dallas, TX, USA) as the positive control. Proteins samples were transferred for 30 min onto Immobilin-FL PVDF membranes (Millipore, Billerica, MA, USA) and membranes scanned to quantify the total protein transferred using a Bio-Rad Gel Doc™ XR+ (Bio-Rad Laboratories, Hercules, CA, USA). Membranes were then blocked for 1 h with 5% skim milk powder/10% Tris-buffered saline with 0.1% Tween 20 (TBST) at room temperature. After blocking, membranes were incubated overnight at 4 °C with primary antibodies for AGAT (1:100; Atlas Antibodies HPA026077, Bromma, Sweden), GAMT (1:1000; Monash Antibody Technologies Facility, Melbourne, Australia); Creatine kinase B type (1:5000; Abcam BBCK ab92452, Cambridge, UK) or creatine kinase u-type mitochondrial (1:1000; Atlas CKMT1A HPA043491) in 5% skim milk powder/TBST.

Membranes were incubated for 1 h with fluorescent secondary antibodies (Anti-Mouse IgG (H+L) Daylight^TM^ 680 Conjugate or Anti-Rabbit IgG (H+L) Daylight^TM^ 800 Conjugate; Cell Signalling Technologies^®^, Danvers, MA, USA). Membranes were exposed on an Odyssey^®^ Infrared Imaging System (LI-COR Biosciences, Lincoln, NE, USA) and individual protein band optical densities were determined using Image Studio Lite software (V5.2.5; LI-COR Biosciences, Lincoln, Nebraska, USA). Optical densities for each sample were quantified and then normalized to the total protein transferred onto the blot for that sample [49]. To account for any inter-blot variability, results were then normalized further using the optical density generated by an internal control (healthy term human placenta sample) run on each blot. Data is expressed relative to the control cohort.

### 4.3. Statistical Analysis

All data were assessed for normality using the Shapiro-Wilk test. Population characteristics (maternal age, parity, gestational age, mode of delivery, baby sex, birth weight and placenta weight) were tabulated. Data are presented as mean ± SD if normally distributed or median and interquartile range (IQR) if non-parametric. Potential confounders, including maternal age, mode of delivery, birth weight and placental weight were assessed with univariate analysis for each of the outcome variables prior to group comparisons being completed. None of these factors were independently associated with changes in outcomes measures. Statistical differences in placental creatine and GAA content, mRNA and protein expression between groups were established with either t-tests or Wilcoxon rank-sum, as appropriate. Baby sex was considered as a covariate for each of these analyses. Correlations between creatine and GAA content, mRNA and protein expression data, with maternal characteristics and birth outcomes were determined using the Spearman rank correlation coefficient. *p* ≤ 0.025 was considered statistically significant for direct comparisons and *p* ≤ 0.004 for correlations after a Benjamini-Hochberg adjustment for false-positives due to multiple comparisons. All analyses were undertaken using SPSS^®^ (Version 23, IBM Corporation, 2015, Armonk, NY, USA).

## 5. Conclusions

Placentae from pregnancies affected by early-onset PE have increased total creatine content compared to normotensive controls. Although the current study did not identify changes to protein abundance, the metabolic stress of PE induced gene expression increases in creatine synthesizing enzymes and cytosolic creatine kinase. These initial observations suggest that the PE placenta may adapt to perturbations in oxygen delivery and reduced ATP production at the site of the mitochondria by heightening its reliance on the creatine kinase circuit to stabilize bioenergetics. These processes should be further explored in the context of mitochondrial and endoplasmic reticulum stress, particularly AMPK activation, which has been shown in other tissues to up-regulate creatine metabolism in response to metabolic stress.

## Figures and Tables

**Table 1:** Population Characteristics.

Characteristics	Control (*n* = 20)	PE (*n* = 20)	*p* Value
Maternal Age (years)	33.5 (5.0) ^1^	28.9 (4.4) ^1^	<0.01
Nulliparous	6.0 (30.0) ^2^	15.0 (75.0) ^2^	<0.01
Gestation (weeks)	34.2 (4.1) ^1^	32.7 (4.0) ^1^	NS
Systolic Blood Pressure (mmHg)	<140	162.3 (11.8) ^1^	-
Diastolic Blood Pressure (mmHg)	<90	102.9 (5.9) ^1^	-
Mode of Delivery			
**Vaginal Delivery**	5.0 (25.0) ^2^	5.0 (25.0) ^2^	NS
**C-Section not in Labor**	14.0 (70.0) ^2^	5.0 (25.0) ^2^	<0.01
**C-Section in Labor**	1.0 (5.0) ^2^	10.0 (50.0) ^2^	<0.01
Birth Weight (g)	3140 (2762–3620) ^3^	1443 (1155–3520) ^3^	<0.05
Placental Weight (g)	463.5 (402–830) ^3^	380.0 (323–600) ^3^	<0.05
Baby Sex (male)	10 (50.0) ^2^	14 (70.0) ^2^	NS

^1^ Mean (standard deviation); ^2^ Number (%) total number; ^3^ Median (interquartile range); NS: not significant.

**Table 2:** TaqMan probe Sequences.

	Gene of Interest	TaqMan Probes
Creatine Synthesis	*GATM*	Hs00933793_m1
	*GAMT*	Hs00355745_g1
Creatine Transport	*SLC6A8*	Hs00940515_m1
Creatine Kinases	*BBCK*	Hs00176484_m1
	*CKMT1A*	Hs00179727_m1
Housekeeping	*RN18S1*	Hs03003631_g1

## Supplementary Materials

Supplementary Materials can be found at https://www.mdpi.com/1422-0067/21/3/806/s1.

## Abbreviations

PE	Pre-eclampsia
GAA	Guanidinoacetate
GATM	Gene—arginine:glycine aminotransferase
AGAT	Protein—arginine:glycine aminotransferase
SLC6A8	Gene—creatine transporter
GAMT	Guanidinoacetate methyltransferas
BBCK	Cytosolic creatine kinase
CKMT1A	Mitochondrial creatine kinase
ADP	Adenosine Diphosphate
ATP	Adenosine Triphosphate
I/R	Ischemic-reperfusion
FGR	Fetal Growth Restriction
mRNA	Messenger Ribonucleic Acid
AMPK	AMP-activated protein kinase
PGC-1α	Peroxisome activated receptor γ coactivator-1 alpha
ERRα	Estrogen-related receptor α
HIF-2α	Hypoxia-inducible factor 2 α
