# Supplementary material for: The Effects of Early-Onset Pre-Eclampsia on Placental Creatine Metabolism in the Third Trimester
Source: Int J Mol Sci. 2020 Jan 26;21(3):806. doi: 10.3390/ijms21030806 (PMC7036877; doi:10.3390/ijms21030806)
Supplement: Supplementary file 1 [file ijms-21-00806-s001.pdf]

Supplementary Materials:

Supplementary information 1:
The following layout was used for each AGAT and GAMT westerns. Control samples are highlighted in red and PE samples in black. Please note, a third cohort of FGR samples were also run on these gels (sample IDs in blue). These data are a part of a subsequent study.

Table S1. Sample Order of Western Blot Gels for AGAT and GAMT Analysis

**Gel One**		**Gel Two**		**Gel Three**	
**Lane**	**Samples**	**Lane**	**Samples**	**Lane**	**Samples**
**1**	*ladder*	**1**	*ladder*	**1**	*ladder*
**2**	I18	**2**	N11	**2**	12862
**3**	7845	**3**	I24	**3**	N18
**4**	8027	**4**	PE-A	**4**	PE-14
**5**	8193	**5**	PE-12	**5**	I28
**6**	I25	**6**	I10	**6**	N25
**7**	8212	**7**	PE-9	**7**	N10
**8**	I26	**8**	N14	**8**	PE-C
**9**	8008	**9**	I27	**9**	8160
**10**	I12	**10**	I14	**10**	PE-E
**11**	PE-6	**11**	8348	**11**	N9
**12**	N6	**12**	N24	**12**	PE-D
**13**	N23	**13**	8059	**13**	N3
**14**	8187	**14**	N69	**14**	I21
**15**	PE-13	**15**	I30	**15**	I11
**16**	N15	**16**	PE-16	**16**	I1
**17**	I19	**17**	I3	**17**	I34
**18**	N1	**18**	I17	**18**	*Ladder*
**19**	N19	**19**	8107	**19**	-
**20**	*Ladder*	**20**	*Ladder*	**20**	*Brain lysate*
**21**	-	**21**	-	**21**	-
**22**	*Control Placenta*	**22**	*Control Placenta*	**22**	*Control Placenta*
**23**	-	**23**	-	**23**	-
**24**	*Liver lysate*	**24**	*Liver lysate*	**24**	*ladder*
**25**	-	**25**	-	**25**	
**26**	*ladder*	**26**	*ladder*	**26**	

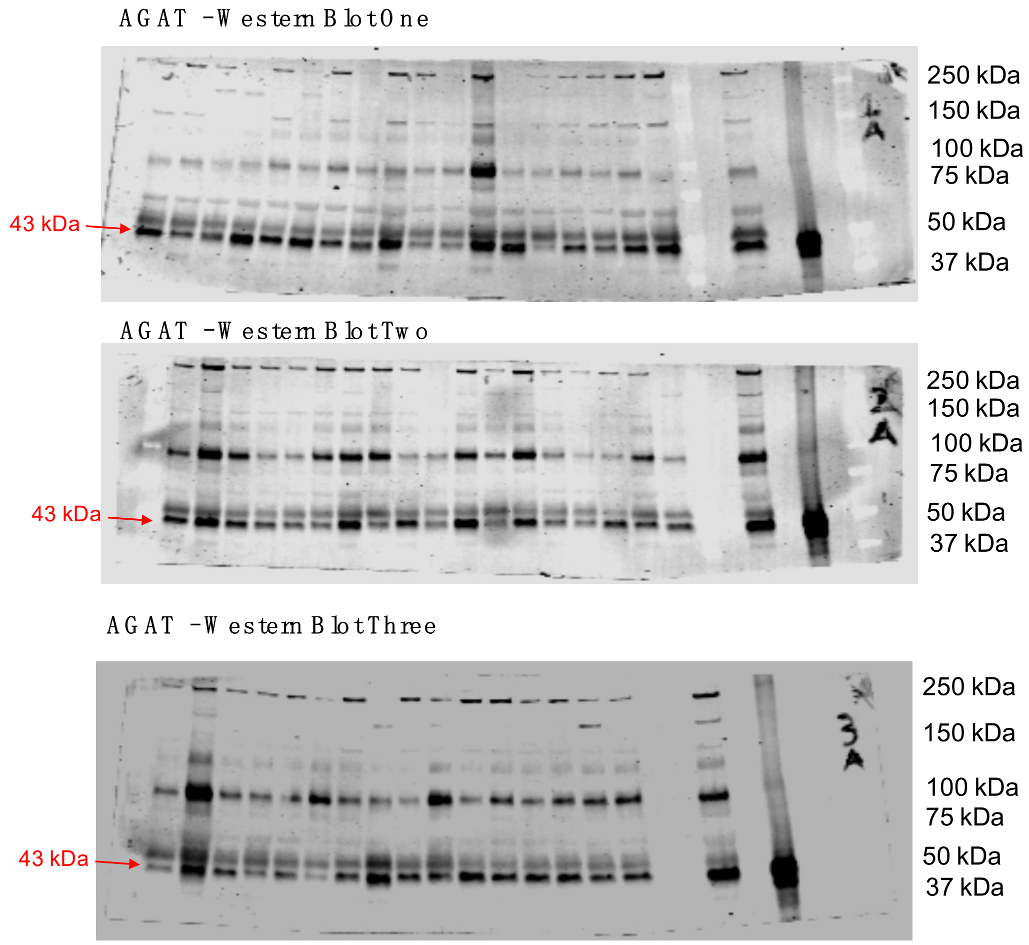

Figure S1. AGAT Western Blots

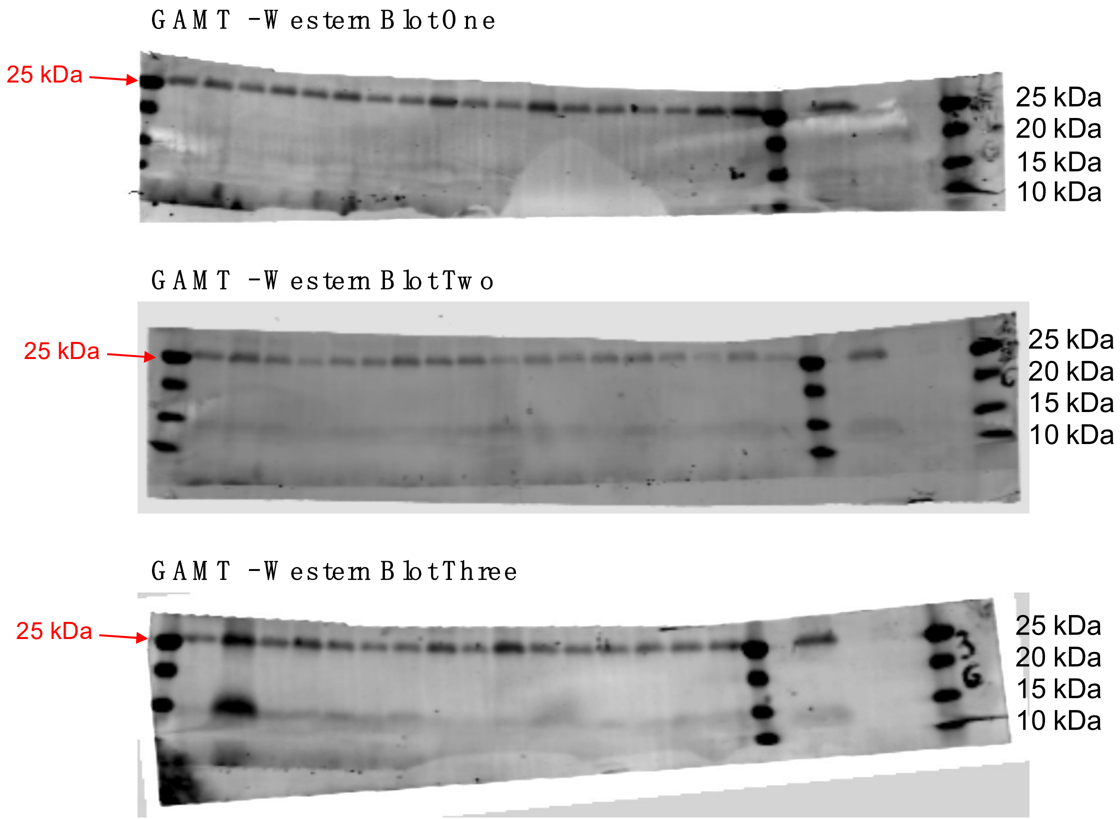

Figure S2. GAMT Western Blots

Supplementary information 2:

The following layout was used for each BBCK and CKMT1A westerns. Control samples are highlighted in red and PE samples in black. Please note, a third cohort of FGR samples were also run on these gels (sample IDs in blue). These data are a part of a subsequent study.

Table S2. Sample Order of Western Blot Gels for BBCK and CKMT1A Analysis

Gel One		Gel Two		Gel Three	
**Lane**	**Samples**	**Lane**	**Samples**	**Lane**	**Samples**
**1**	*ladder*	**1**	*ladder*	**1**	*ladder*
**2**	I18	**2**	N11	**2**	12862
**3**	7845	**3**	I24	**3**	N18
**4**	8027	**4**	PE-A	**4**	PE-14
**5**	8193	**5**	PE-12	**5**	I28
**6**	I25	**6**	I10	**6**	N25
**7**	8212	**7**	PE-9	**7**	N10
**8**	I26	**8**	N14	**8**	PE-C
**9**	8008	**9**	I27	**9**	8160
**10**	I12	**10**	I14	**10**	PE-E
**11**	PE-6	**11**	8348	**11**	N9
**12**	N6	**12**	N24	**12**	PE-D
**13**	N23	**13**	8059	**13**	N3
**14**	8187	**14**	N69	**14**	I21
**15**	PE-13	**15**	I30	**15**	I11
**16**	N15	**16**	PE-16	**16**	I1
**17**	I19	**17**	I3	**17**	I34
**18**	N1	**18**	I17	**18**	*Ladder*
**19**	N19	**19**	8107	**19**	-
**20**	*Ladder*	**20**	*Ladder*	**20**	*Control Placenta*
**21**	-	**21**	-	**21**	-
**22**	*Control Placenta*	**22**	*Control Placenta*	**22**	*Brain Lysate*
**23**	-	**23**	-	**23**	-
**24**	*Brain lysate*	**24**	*Brain lysate*	**24**	*ladder*
**25**	-	**25**	-	**25**	
**26**	*ladder*	**26**	*ladder*	**26**	

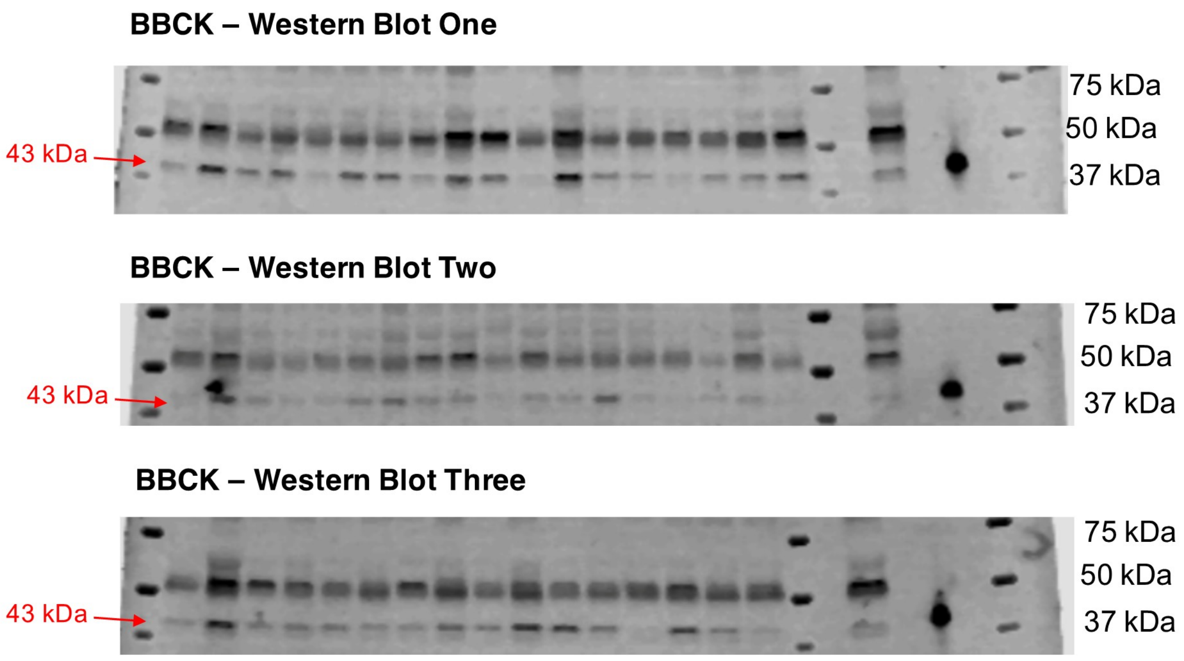

Figure S3. BBCK Western Blots

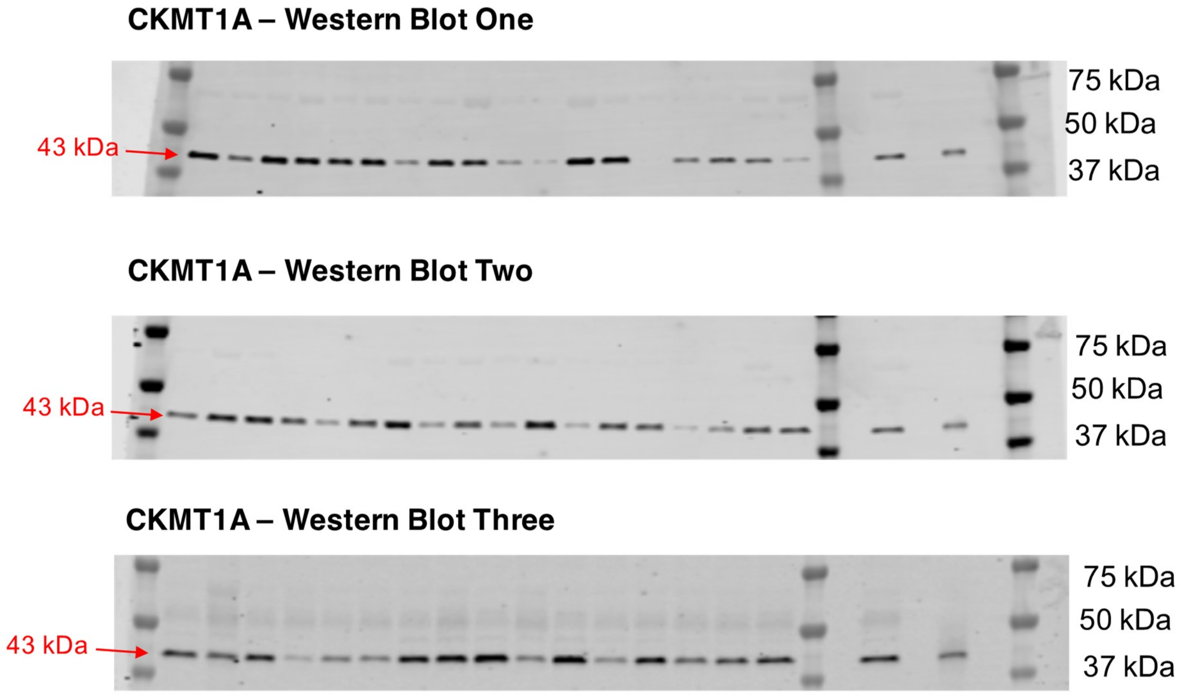

Figure S4. CKMT1A Western Blots
